# Coupled Effect of Nutritional Food Molecules and *Lactobacillus reuteri* Surface Protein Interaction on the Bacterial Gastrointestinal Tolerance

**DOI:** 10.3390/foods13223685

**Published:** 2024-11-19

**Authors:** Ao Zhang, Mingjuan Ou, Peng Wu, Kaige Zheng, Haiqian Zhang, Yixing Yu, Yuxing Guo, Tao Zhang, Daodong Pan, Zhen Wu

**Affiliations:** 1 State Key Laboratory for Managing Biotic and Chemical Threats to the Quality and Safety of Agro-Products, Key Laboratory for Food Microbiology and Nutrition of Zhejiang Province, College of Food Science and Engineering, Ningbo University, Ningbo 315211, China; 2401390161@nub.edu.cn (A.Z.); 236000582@nbu.edu.cn (P.W.); 2311390026@nbu.edu.cn (K.Z.); 2311390095@nbu.edu.cn (H.Z.); 2111085040@nbu.edu.cn (Y.Y.); zhangtao@nbu.edu.cn (T.Z.); daodongpan@163.com (D.P.); 2 School of Food Science & Pharmaceutical Engineering, Nanjing Normal University, Nanjing 210097, China; guoyuxing1981@163.com

**Keywords:** *Lactobacillus reuteri* DSM 8533, intestinal nutrition molecules, LPxTG protein, gastrointestinal tolerance, docking

## Abstract

*Lactobacillus reuteri,* which is present in fermented foods, can produce LPxTG motif proteins (LMPs) to help the strain resist gastrointestinal fluid environmental stress and enhance the adherence and colonizing properties. Intestinal nutrient small molecules can interact with LMPs and cooperate with *Lactobacillus* to exert probiotic effects in the host intestine. However, the mechanism of their correlation with gastrointestinal tolerance needs to be further studied. In this study, different kinds of nutritional food molecules, such as intestinal phenols, sugars, and acids, were screened and the interaction between the LPxTG proteins and small molecules was explored via the molecular docking approach. The docking results showed that phenols and oligosaccharides were more likely to bind to the LPxTG protein (B3XKV5), with the benzene ring, phenolic hydroxyl group, and glycosidic bond in the small molecule more easily binding to the active site of B3XKV5. Furthermore, the gastrointestinal tolerance was enhanced under the rutin, myricetin, quercetin phenols, and stachyose-treated *L. reuteri* strain groups, especially the phenol group, which revealed the relationship between the molecular interaction of the strain with the small molecules and strain tolerance mechanism. All the findings illustrated the gastrointestinal tolerance escape effect of the *Lactobacillus* strain under enriched intestinal nutrient small molecular conditions, and they also provide insight regarding the small molecules for the *Lactobacillus* strain under abnormal growth environments.

## 1. Introduction

*Lactobacillus*, commonly found in various fermented foods, must withstand multiple environmental stresses in the gastrointestinal tract (GIT) after ingestion, including exposure to gastric acid, pepsin, bile salts, and trypsin. Despite these challenges, certain probiotic strains can successfully colonize the GIT, where they assist in the absorption of digested nutrients, enhance nutrient bioavailability, regulate immune function, balance the intestinal microbiota, lower cholesterol levels, exhibit antioxidant properties, and help to reduce blood pressure [1]. Additionally, these strains can interact with food molecules to exert probiotic effects, contributing to the maintenance of the host’s intestinal ecological balance [2].

Adhesion and colonization are crucial indicators of the probiotic potential of lactic acid bacteria (LAB) in the gastrointestinal tract. Strong tolerance enhances LAB’s ability to adhere to and colonize the host’s intestines, with both processes synergistically contributing to the overall probiotic effects [3,4]. The tolerance and adhesion capabilities of the *Lactobacillus* strains in the gastrointestinal environment are closely associated with their surface layer proteins (SLPs) [5]. In some Gram-positive bacteria, surface proteins contain a specific C-terminal cell wall sorting signal anchored to the LPxTG motif, which includes an LPxTG sequence, a hydrophobic domain, and a positively charged tail. These proteins are known as LPxTG motif proteins (LMPs) [6]. LMPs not only serve as protective agents in hostile gastrointestinal environments, functioning as molecular sieves and ion traps, but also help to maintain cell morphology, resist adverse conditions, and play a pivotal role in the adhesion and colonization of *Lactobacillus* [7]. Furthermore, LMPs facilitate the transport and delivery of substances through interactions with extracellular nutrient molecules. Moreover, studies have demonstrated that these proteins play a crucial role in the exchange of materials and signals between *Lactobacillus*, the intestinal microbiota, intestinal epithelial cells, and the host [8].

Nutritionally active food molecules, such as small peptides, oligopeptides, oligosaccharides, organic acids, vitamins, minerals, and phenolic compounds, possess both biological and nutritional properties [9,10]. In the gastrointestinal environment, some intestinal nutrients with prebiotic potential cannot be directly decomposed or utilized by the human body. However, lactic acid bacteria colonized in the intestine can help the host to absorb and utilize these nutrient molecules. The two synergistically exert probiotic effects through interaction [11]. Oligosaccharides, as a potent carbon source, can be utilized by lactic acid bacteria (LAB) during their passage through the digestive tract, promoting their survival and facilitating colonization. This confers a competitive advantage to probiotics in the gut, ultimately benefiting the host [12,13]. However, the precise mechanisms through which food molecules interact with probiotic *Lactobacillus* strains remain unclear. Further research is needed to elucidate how these molecules interact with bacteria and whether surface proteins of the strains play a role in binding to these molecules.

AutoDock Vina is widely used for the flexible docking of small-molecule ligands with receptor proteins and supports batch processing, making it highly versatile. Its application potential in the field of food science, including areas such as nutrition and food safety, has garnered increasing attention. For example, Liu et al. (2022) employed molecular docking to demonstrate that the interaction between whey protein and polyphenols enhances the functional properties of whey protein [14]. Additionally, novel anti-inflammatory peptides from broccoli were identified through peptidomic analysis combined with molecular docking under co-fermentation conditions with *L. plantarum A3* and *Rhamnose ATCC7469* [15]. In recent years, prebiotics derived from small-intestinal-nutrient molecules have become a research focus in the field of gut health. However, the relationship between these nutritional small molecules and probiotics, particularly in the context of molecular docking, remains poorly understood and requires further investigation. Moreover, how these small molecules interact with LPxTG motif proteins (LMPs) and their connection to the gastrointestinal tolerance mechanisms of *Lactobacillus* strains also demand deeper exploration. *Lactobacillus reuteri* is a well-studied probiotic that colonizes the human gastrointestinal tract and strengthens the intestinal barrier. Notably, intestinal nutritional molecules can support *L. reuteri* colonization and reshape the composition of the host’s symbiotic microbiota [16]. In our previous study, LPxTG protein B3XKV5 was found to be up-regulated through transcriptome analysis [17]. We analyzed that LMPs can not only help the colonization of lactic acid bacteria in the intestine but also further help the host to absorb and utilize the nutritional small molecules with probiotic potential. The target LPxTG structural protein was successfully purified using a recombinant expression system in *E. coli*. To further investigate the interaction between the LPxTG protein and food-derived molecules, molecular docking will be employed to assess the binding properties of protein B3XKV5 with phenolic compounds, carbohydrates, and acidic small molecules from intestinal nutrients. This approach will help to identify representative nutrient molecules. Through structure–activity analysis, we aim to explore the relationship between molecular interactions and the tolerance mechanism of *L. reuteri* SH23, with the goal of confirming these findings in this study.

## 2. Materials and Methods

### 2.1. Reagents and Strains

*Lactobacillus reuteri* DSM 8533 was cultured in MRS broth (MRS) at 37 °C. *E. coli* BL21(DE3) competent cells were purchased from Sangon Biotech (Sangon, Shanghai, China). PCR products were cloned using the pEASY^®^-T1 cloning kit, which was purchased from Transgen Biotech (Transgen Biotech, Beijing, China), where cloning was performed in Man–Rogosa–Sharpe (MRS) medium at 37 °C for 24 h and stored in 30% glycerol (−80 °C) for further use.

### 2.2. Structure Prediction and Homology Modeling of Protein B3XKV5

The transmembrane structure of the target protein was predicted using the biological online software tool TMHMM Server v.2.0 (http://services.healthtech.dtu.dk, accessed on 18 August 2024). Because the three-dimensional structure of B3XKV5 has not been resolved, the three-dimensional structure of B3XKV5 was constructed by homology modeling method based on the Swiss model repository (http://swissmodel.expasy.org/repository/, accessed on 22 August 2024) according to the methods of Blanes-Mira with some modifications [18].

### 2.3. AutoDock Vina Semi-Flexible Batch Docking

AutoDock Tools (v.1.5.7) were used to process the prepared receptor protein PDB file and ligand small molecule PDB file, and the PDBQT format file was generated after hydrogenation, water removal, metal ion removal, charge addition, and torsion bond confirmation. AutoDock Vina (v.1.2.3) was used to dock the PDBQT of the prepared receptor with all the selected nutritional food molecules in batches. For the visualization and analyzing the docking results, PyMOL (v.2.5.7) and Discovery Studio were used. Firstly, the PDBQT file was generated by batch docking, and the original protein receptor PDB file prepared in the early stage was imported into PyMOL and at the same time in the visual processing. Secondly, the hydrogen bond was displayed, the appropriate angle was adjusted, and the hydrogen bond was displayed using instructions. Finally, the bonding position amino acid residue numbers were ILE-3984, ALA-3989, and ASN-4015, the protein docking site was named, and hydrogen bond length was displayed in the docking visual 3D processing results.

### 2.4. Cloning, Expression, and Purification of LMP

The surface LPxTG protein gene (B3XKV5) of *Lactobacillus reuteri* DSM 8533 consists of a 1710 bp open reading frame encoding a polypeptide of 570 amino acids (Uniprot ID: B3XKV5). The cloning, expression, and purification of B3XKV5 refer to the method of Xu et al. [17]. The amplified target genes were purified by agarose gel DNA purification kit from Transgen Biotech (Transgen, Beijing, China). After that, they were digested with EcoRI and XhoLI restriction sites (from Takara Biomedical Technology, Beijing, China), cloned into pET-28a, expressed in *E. coli* BL21 (DE3), and grown in Luria–Bertani (LB) medium. Finally, LMPs were purified by ProteinIso^®^ Ni-IDA Resin HisTrap column (TransGen, Beijing, China) and eluted with 100 mM imidazole buffer (100 mM imidazole, 10 mM Tris, 0.3 M NaCl, and 50 mM NaH_2_PO_4_; pH = 8.0).

### 2.5. Determination of the Effect of B3XKV5 and Small Molecules on the Growth Activity of the Strain

After *Lactobacillus reuteri* DSM 8533 was activated to the third generation, the bacteria were washed three times with sterile PBS and then diluted with PBS to adjust the bacterial solution to OD_600_ = 1.00 ± 0.05, and the absorbance of OD_600_ was measured by a microplate reader. After that, three different treated groups were prepared with single nutritional food molecules, B3XKV5 protein, and mixed molecule/B3XKV5; the final concentrations of each small molecule were 0.05 mg/mL, 0.1 mg/mL, 0.2 mg/mL, and 0.4 mg/mL, and the protein was added to the final concentration of 0.2 mg/mL. After that, the bacterial solution sample with OD_600_ = 1 was inoculated into each MRS broth containing the target small molecule and/or B3XKV5 protein at a concentration of (*v*/*v*) and cultured at 37 °C. The absorbance (OD_600_) of the bacterial solution was measured every two hours, and the data were recorded for the growth curve generation.

### 2.6. The Effect of Interaction Between B3XKV5 and Small Molecules on the Tolerance of Strains to the Artificial Gastrointestinal Fluid

The artificial gastric fluid was prepared as follows: 0.5% NaCl (0.4822 g) was resolved in 100 mL water, the pH of the solution was adjusted to 2 with high concentration of HCl, autoclaved at 121 °C for 15 min, and then pepsin was added to the final concentration of 3 g/L (sterile operation through 0.22 μm filter membrane). Artificial intestinal fluid preparation: 0.5% NaCl (0.4822 g) + 0.5% bovine bile salt (0.4822 g) + 100 mL water, the pH of the solution was adjusted to 8 with 0.5M NaOH, autoclaved at 121 °C for 15 min, and then trypsin was added to the final concentration of 3 g/L (sterile operation through 0.22 μm filter membrane).

Three different groups were prepared for the strain tolerance analysis under the artificial gastrointestinal fluid; bacterial solution (OD_600_ = 1.00 ± 0.05) without the fluid treatment was defined as the control group. For the target-small-molecule-treated group, bacteria with the same OD value were first put in the 10 mL supernatant gastric juice and then the target small molecules and the target LPxTG protein in each experimental group were added (small molecules added to the final concentration of 0.2 mg/mL; the target LPxTG protein added to the final concentration of 0.2 mg/mL). After that, all the treated groups were cultured in a 37.5 °C environment for 2 h. The liable bacteria were numbered with the coating plate method after 48 h at 37 °C.

### 2.7. Gene Expression (B3XKV5) in Simulated Gastrointestinal Fluid Environment

*Lactobacillus reuteri* DSM 8533 was inoculated in MRS medium at 37 °C as the control group. The experimental group followed the methods of the tolerance test in the artificial gastrointestinal fluid. Then, the cells were collected for RNA extraction using the Magen kit (Magen Biotech Co., Ltd., Guangzhou, China). Next, the extracted RNA was reversely transcribed using the kit (TranScript All-in-One First-Strand cDNA Synthesis SuperMix for qPCR, TransGen Biotech, Beijing, China). Finally, 16S rRNA was used as a reference gene. The primers used in this study were designed using Primer Premier 6.0 (Premier Biosoft International, San Francisco, USA).

Gene-specific primers as follows: 16SrRNA gene, forward (5′-TACCGCATAACAACTTGGACC-3′), reverse (5′-GCCGAAGGCTTTCACATCA-3′); B3XKV5 gene, forward (5′-AACAAGAAAAGCAACAGGTT-3′), reverse (5′-CTATTTCTCTCGTTTCTTCT-3′).

Quantitative real time-PCR (qRT-PCR) was performed according to the kit guidelines (TransStart Tip Green qPCR SuperMix, TransGen Biotech, Beijing, China).

### 2.8. B3XKV5 Small-Molecule Circular Dichroism Determination

The concentration of the purified B3XKV5 protein was diluted to a final concentration of 0.2 mg/mL. The target small food molecules rutin, myricetin, stachyose, and quercetin were added to the protein solution to a final concentration of 0.2 mg/mL in sterile water. At 25 °C, the solution was placed in a 0.1 cm quartz cuvette, and the 190–260 nm band was scanned by a circular dichroism spectrometer. The scanning speed was 100 nm/min. The sterilized deionized water was used as the measurement baseline, and then the sample circular dichroism test was carried out.

### 2.9. AI-2 Signal Intensity Determination Based on Molecular Interaction

The method adopted and slightly modified according to the Taga ME bioluminescence [19]. *L. reuteri* DSM 8533 was cultured and activated in MRS medium at 37 °C for two generations, and then the bacteria were washed and the OD value was adjusted to OD_600_ = 1.00 ± 0.05; 1% (*v*/*v*) was inoculated into the new MRS medium. Set control group and experimental group. After that, bacterial cultures with different molecule additions were centrifuged at 8000 rpm, 10 min. In the experimental group, each nutrient small molecule, B3XKV5 protein, and the combination of each nutrient small molecule and B3XKV5 protein were added separately to the inoculated MRS medium (final concentration was 200 ug/mL) and cultured at 37 °C for 12 h. The bacterial solution with different molecules was centrifuged at 8000 rpm for 10 min, and the supernatant was collected by 0.22 μm filter membrane for AI-2 signal intensity determination.

The AI-2 reporter strain *Vibrio harveyi* BB170 was cultured in AB medium at 30 °C for 6 h, and then the culture was inoculated in fresh AB medium at 1:5000. The cell-free medium and culture were mixed at a ratio of 1:9 and cultured at 30 °C on a shaker at a shake rate of 150 r/min. During the culture process, the fluorescence intensity was measured every 3 h using a multifunctional microplate instrument (infinite M200 PRO, Tecan, CH, Mennedorf, Switzerland), and the data were recorded to draw the fluorescence intensity change curve. The excitation and emission wavelengths are 485 nm and 538 nm, respectively.

### 2.10. Statistical Analysis

All statistical analyses were performed using one-way ANOVA via 26 SPSS software for significance analysis of variance, and multiple comparisons were performed using LSD and Duncan multiple comparisons. The *p*-value < 0.05 was considered statistically significant. All values were expressed as mean ± standard deviation (X ± SD).

## 3. Results

### 3.1. B3XKV5 Modeling Information and Transmembrane Structure Prediction

**Figure 1:**
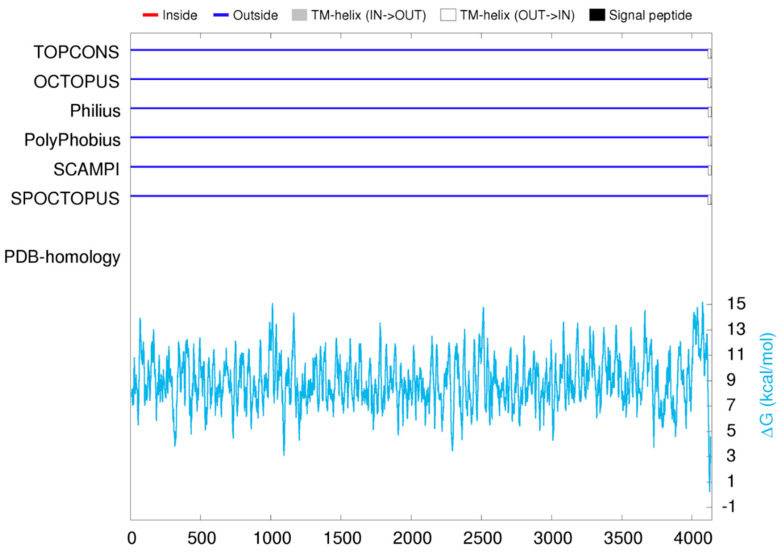
Transmembrane structure prediction of the surface protein B3XKV5 results map.

As shown in Table 1, the QMEAN modeled by the Swiss model was 0.55 ± 0.08. The template protein is monomeric and does not contain ligands. The transmembrane structure prediction results show that B3XKV5 exists outside the cell membrane and has no intracellular structure (Figure 1). As shown in Table 2, the modeling template is 4ng0.1.A, and the Seq Identity is 38.30 with a similarity of 0.39 according to the X-ray method, which is a surface adhesion protein of *L. reuteri*.

### 3.2. Docking Affinity of B3XKV5 with the Nutritional Food Molecules

**Figure 2:**
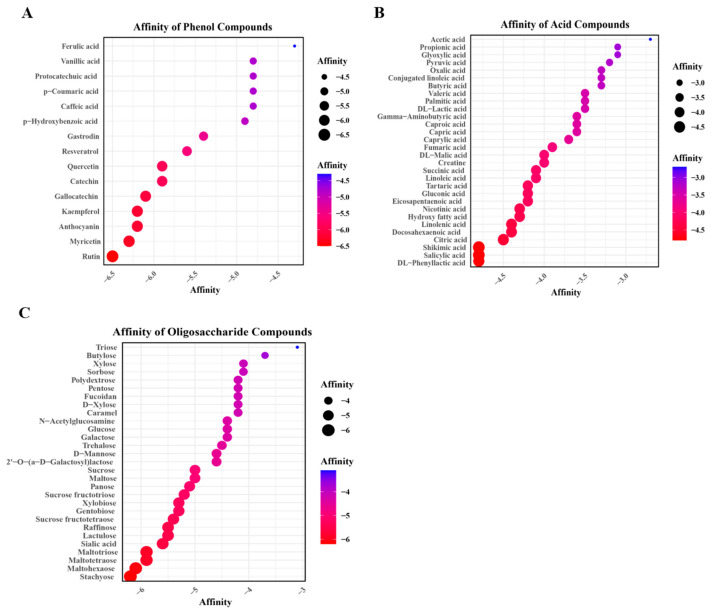
Bubble diagram of batch docking results of small nutrient molecules with B3XKV5. (**A**) Batch docking affinity results of phenolic small molecules with B3XKV5; (**B**) batch docking affinity results of acid small molecules with B3XKV5; (**C**) the affinity results of batch docking of oligosaccharide small molecules with B3XKV5.

In this study, three phenols, oligosaccharides, and organic acids were screened as the nutritional food molecules. As shown in Figure 2A, nine phenolic small molecules have a binding affinity value lower than −5 Kcal/mol with B3XKV5 as affinity lower than −5 Kcal/mol is considered to be a stable binding. Among them, the binding effects of rutin, myricetin, and quercetin as ligands to receptor B3XKV5 protein were the best, and the affinity values were −6.5 Kcal/mol, −6.3 Kcal/mol, and −5.9 Kcal/mol, respectively. The binding stability of ferulic acid and caffeic acid was not as expected, the values being −4.3 Kcal/mol and −4.8 Kcal/mol, respectively. Furthermore, the secondary structures of rutin and myricetin, as phenolic substances, presented stronger affinity to the B3XKV5 protein, as shown in Table 3. As for ferulic acid and caffeic acid, the results indicated the weakest affinity, as found also in the binding.

As shown in Figure 2B, the affinity values of all the acid molecules with B3XKV5 are lower than −5 Kcal/mol, which is the worst of the three types of small molecules. Regarding the strongest affinity values of phenyllactic acid and salicylic acid, the docking affinity values were −4.8 Kcal/mol and −4.8 Kcal/mol. The weakest affinity was observed in acetic acid and propionic acid. Meanwhile, all four representative acids contain carboxyl groups (Table 4). As for the binding strength of the oligosaccharides with the B3XKV5 protein, stachyose has the best bond quality in oligosaccharides, and the affinity was −6.2 Kcal/mol (Figure 2C). Triose was the least effective small molecule with an affinity of −3.1 Kcal/mol. It is worth noting that the strength of maltohexaose, maltotetraose, maltotriose, and maltose gradually decreased with reductions in length. In Table 5, stachyose, maltohexaose, maltotetraose, maltotriose, and maltose all contain glycosidic bonds.

### 3.3. Visualization of Docking Results

**Figure 3:**
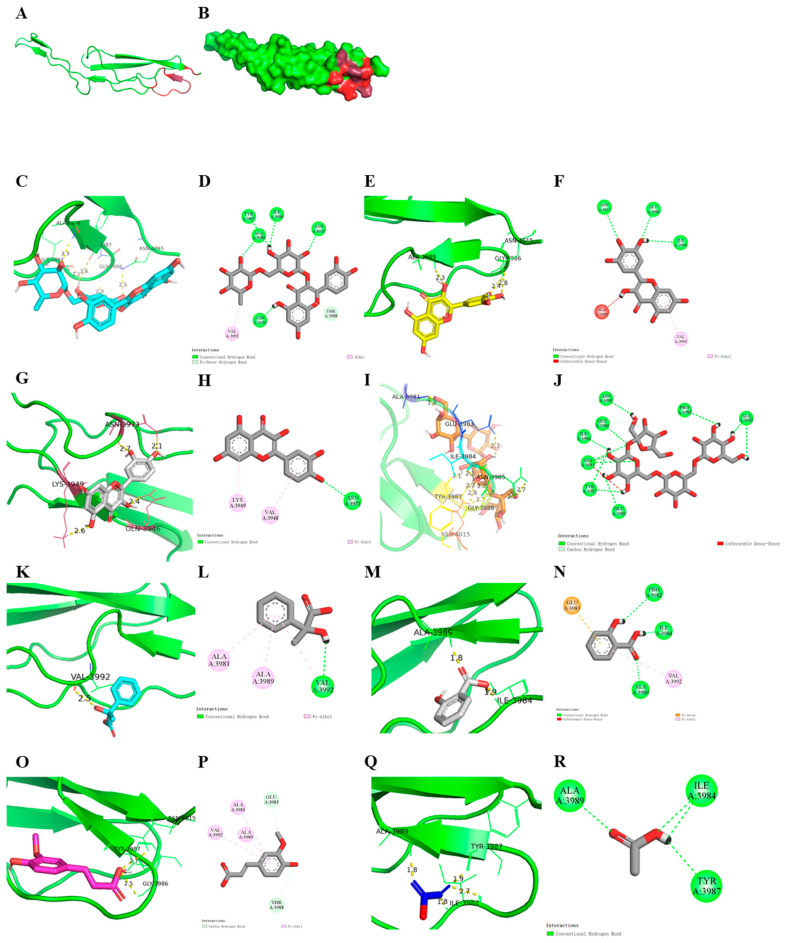
3D and 2D visualizations of docking results. (**A**,**B**) Protein 3D model of the B3XKV5, red area for the active pocket; (**C**,**D**) binding status of rutin with B3XKV5; (**E**,**F**) binding status of myricetin with B3XKV5; (**G**,**H**) binding status of quercetin with B3XKV5; (**I**,**J**) binding status of stachyose with B3XKV5; (**K**,**L**) binding status of DL-3-phenyllactic acid with B3XKV5; (**M**,**N**) binding status of salicylic acid with B3XKV5; (**O**,**P**) binding status of ferulic acid with B3XKV5; (**Q**,**R**) binding status of acetic acid with B3XKV5. The green dotted line represents the traditional hydrogen bond; The pink dotted line represents the hydrophobic force; The orange dotted line represents the electrostatic force.

Due to the large docking volume, rutin, myricetin, quercetin, and ferulic acid were selected as the potential phenolic small molecules and stachyose as the potential oligosaccharide small molecules. Phenyllactic acid, salicylic acid, and acetic acid were selected for the analysis of the binding posture, amino acid name and number labeling of the active sites, active pocket confirmation, and structure–activity relationships of the small molecules. The results indicated that most of the hydrogen bond binding sites formed by the small molecule with the B3XKV5 protein were related to amino acid residues ILE-3984, ALA-3989, and ASN-4015. The active binding pockets of protein B3XKV5 are shown in Figure 3A,B, and the 3D and 2D docking results of rutin, myricetin, and quercetin with B3XKV5 are shown in Figure 3C–H, which demonstrated that phenolic small molecules can form hydrogen bonds with B3XKV5.

The docking results of stachyose and B3XKV5 are shown in Figure 3I,J. Evidently, the glycosidic bond of stachyose forms multiple hydrogen bonds with the active site ILE-3984 and ASN-4015 amino acid residues. Regarding phenyllactic acid and salicylic acid, the hydroxyl group in phenyl lactic acid formed hydrogen bonds with VAL-3992 amino acid residues, and the benzene ring formed electrostatic forces with ALA-3981 and ALA-3989 (Figure 3L). The benzene ring in salicylic acid forms a hydrophobic force with the amino acid residue at position GLU-3983, the carboxyl group forms a hydrogen bond with ALA-3989 and ILE-3984, and the phenolic hydroxyl group forms a hydrogen bond with PRO-3982 (Figure 3N).

Regarding ferulic acid and acetic acid, the carboxyl group in ferulic acid forms hydrogen bonds with the amino acid residues at positions TYR-3987 and GLY-3986 (Figure 3O). The carboxyl group in acetic acid forms hydrogen bonds with amino acid residues ALA-3989, ILE-3984, and TYR-3987 (Figure 3Q,R). It is easier to determine the interaction rules from the structures and characteristics of small molecules. However, the binding effect of molecules with B3XKV5 was not so strong, so rutin, myricetin, quercetin, and stachyose were selected as the target nutritional small molecules for further verification experiments.

### 3.4. The Effect of Small Molecules and LPxTG Protein on the Growth of the Strain

**Figure 4:**
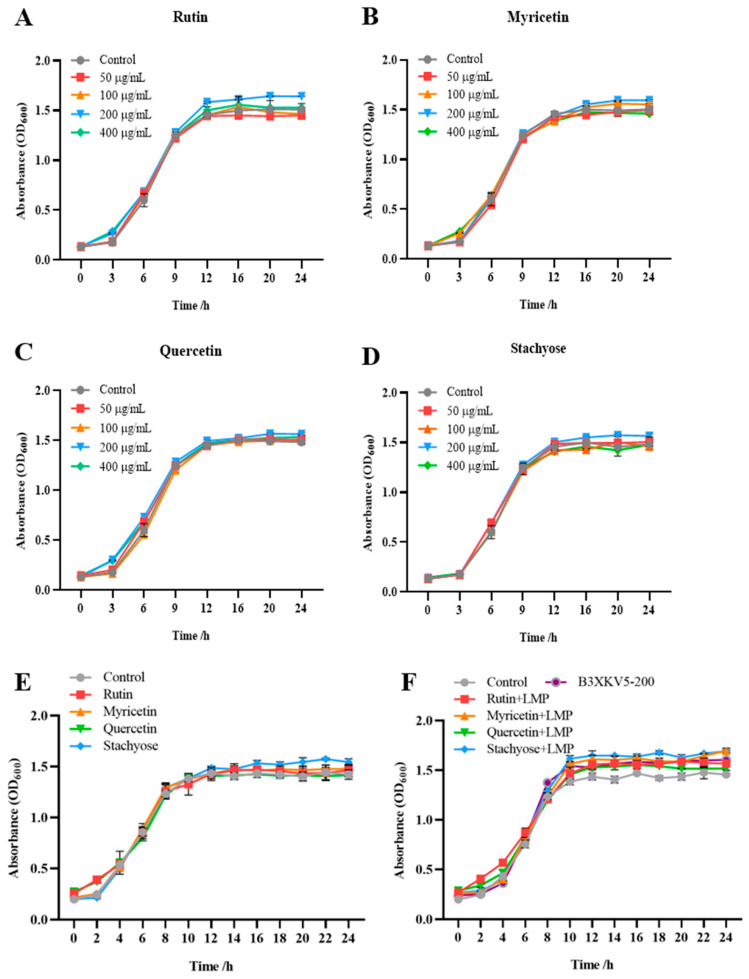
The coupled effect of nutritional food molecules and B3XKV5 protein interaction on the growth of *L. reuteri*. (**A**–**D**) The effect of different concentrations of nutritional food molecules (rutin, myricetin, quercetin, and stachyose) on the growth curve of the strain; (**E**) the effect of the optimum concentration (200 ug/mL) of each nutritional food molecule type (rutin, myricetin, quercetin, and stachyose) on the growth curve of the strain was added separately; (**F**) the effects of various nutritional small molecules (rutin, myricetin, quercetin, and stachyose, 200 ug/mL) on the growth of the strain were combined with B3XKV5 (200 ug/mL) at the optimal concentration.

**Figure 5:**
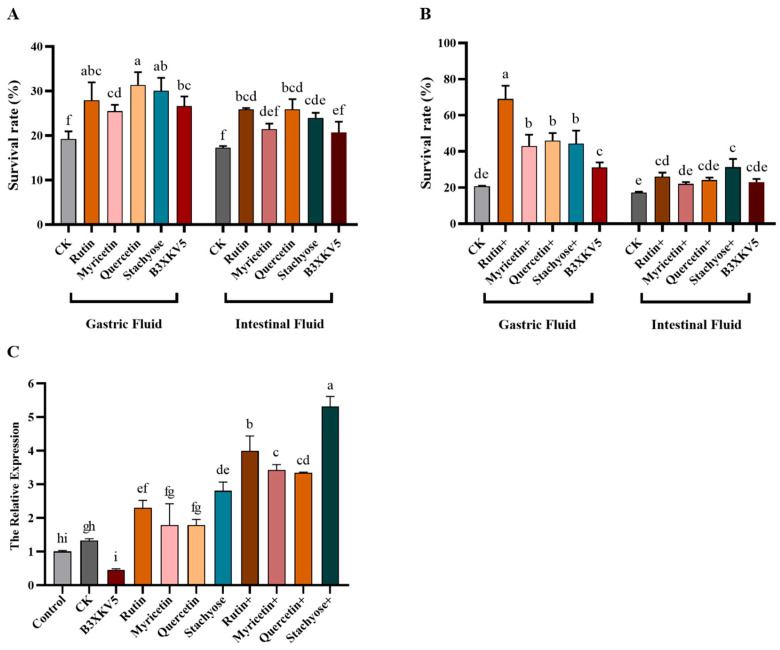
The coupled effect of nutritional food molecules and B3XKV5 protein interaction on the gastrointestinal tolerance of *L. reuteri*. (**A**) The survival rate of *L. reuteri* in gastrointestinal fluid when treated with the nutritional small molecules and B3XKV5 separately; (**B**) the survival rate of *L. reuteri* when treated with nutritional small molecules and B3XKV5 together; (**C**) the expression of B3XKV5 gene in the nutritional small molecules and B3XKV5 single and mixed groups. The same letters of a–i represent no significant difference (*p* > 0.05).

When the concentration of the four small molecules (rutin, myricetin, quercetin, and stachyose) was 200 μg/mL, the absorbance after 12 h was higher than that of the control group without the addition of small molecules (Figure 4). The addition of small molecules had no inhibitory effect on the growth of the strain. Therefore, a 200 μg/mL concentration of each small molecule was selected for further experiments. At the same time, the absorbance value at 10 h was generally higher than that of the single-small-molecule group. The addition of protein caused the growth of the strain to enter a stable period earlier. After 12 h, the combination of stachyose, rutin, and myricetin with better affinity results with B3XKV5 made the absorbance higher than that of the single-B3XKV5 group (Figure 5).

### 3.5. Tolerance Effects of L. reuteri Under Single- and Small-Molecule Combined Groups to Gastrointestinal Fluid

As shown in Figure 5A,B, both small food molecules and proteins can increase the tolerance of the strain to gastrointestinal fluid. The gastric juice treatment group increased from less than 20% to more than 25%. In the group treated with gastric juice and gastrointestinal fluid, quercetin and stachyose had better rescue activity; the combined addition of stachyose–B3XKV5 in the combined treatment group made the survival rate of the strain higher than 40% and 30%, respectively.

Furthermore, the genes of B3XKV5 were also investigated (Figure 5C). The gene expression of B3XKV5 was slightly up-regulated after gastrointestinal fluid treatment, and the addition of four small molecules alone increased the gene expression of B3XKV5. When the strain was pretreated with the B3XKV5 protein, the same result was also found in the rutin and stachyose combination group, which may enhance the tolerance activity of the strain in the gastrointestinal fluid environment.

### 3.6. Signal Intensity (AI-2) Changes in Strain upon Small-Molecule Treatment

**Figure 6:**
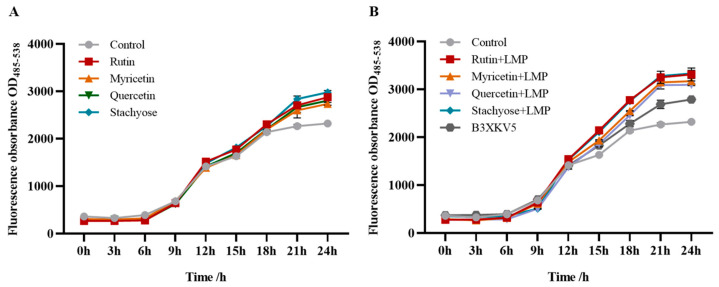
The coupled effect of nutritional small molecules and B3XKV5 on the AI-2 signal intensity of *L. reuteri*. (**A**) The effect of adding each nutrient small molecule alone on the signal intensity of AI-2. of *L. reuteri*; (**B**) the effect of small nutrient molecules combined with B3XKV5 on the signal intensity of AI-2 of the *L. reuteri*.

The addition of small molecules alone enhanced the intensity of the AI-2 signal molecules of the strain (Figure 6A), and the stachyose and rutin groups could produce more AI-2 signal molecules. When combined with B3XKV5, the fluorescence value of the small-molecule–B3XKV5 combined group was higher than that of the small-molecule and single-protein group (Figure 6B). The same trend also occurred regarding the addition of the rutin–B3XKV5 and stachyose–B3XKV5 combined groups, with higher AI-2 signal intensity values of OD_(485–538)_ = 3310 and OD_(485–538)_ = 3330, respectively.

## 4. Discussion

Prebiotics such as rutin, myricetin, quercetin, and stachyose possess potential nutritional values during intestinal digestion, and their appropriate intake positively influences the intestinal colonization of *Lactobacillus*, human biochemical processes, and strain–host interactions [20,21]. However, there is limited research on the combined effects of these small nutritional molecules and bacterial surface proteins on the gastrointestinal tolerance of *L. reuteri*. It remains unclear whether the proteins present on the extracellular surface of *L. reuteri* can facilitate bacterial colonization in the intestine by capturing small nutritional molecules from the external environment in the gastrointestinal tract. To address this, we selected various phenolic compounds, organic acids, and oligosaccharides to dock with B3XKV5, a novel adhesion-related surface protein found in *L. reuteri*, and screened for small molecules exhibiting the best binding effects.

It was observed that the phenolic compounds contained a benzene ring structure and the phenolic hydroxyl groups exhibited a higher affinity for binding to the surface protein B3XKV5. In contrast, the carboxyl groups present in ferulic acid and caffeic acid resulted in unstable binding interactions. Among the acidic molecules examined in this study, phenyllactic acid and salicylic acid demonstrated the strongest binding affinity for B3XKV5, both of which possess benzene ring structures absent in other acidic molecules. The visualization results confirmed that the benzene ring participated in the interaction with B3XKV5, while the phenolic hydroxyl group formed hydrogen bonds with the receptor (Figure 3). This supports the characteristic structure of phenolic compounds, which typically include a benzene ring, with phenyl cations exhibiting high reactivity. Additionally, Zhou [22] confirmed that the phenolic compound apigenin (Api), with its active phenolic hydroxyl group on the benzene ring, possesses a greater capacity for binding to edible docking proteins (EDPs).

Furthermore, both ferulic acid and acetic acid contain carboxyl groups. While ferulic acid possesses a benzene ring structure, the presence of carboxyl groups adversely affects the binding affinity of these small molecules to B3XKV5. Stank et al. (2016) noted that a group of amino acid residues surrounding the binding pocket influence the physical and chemical properties of the active site, ultimately determining its functionality alongside its shape and position within the protein; additionally, residues outside the binding site may also play a role [23]. For instance, the active site of an enzyme typically features a concave shape, enabling amino acid residues to be arranged in an optimal configuration for binding low-molecular-weight compounds. In contrast, the macromolecular binding pocket is usually located on the protein’s surface and tends to be shallow. This observation is consistent with the characteristics of the active pockets identified in this study (Figure 3A,B).

The glycosidic bond of stachyose in oligosaccharides can form hydrogen bonds with the active site of B3XKV5. In contrast, maltohexaose, maltotetraose, maltotriose, and maltose showed a decreasing trend in binding force, which may be due to the decrease in the α-D-glucopyranose unit and the decrease in the α-1,4-glycosidic bond, which shortened the chain structures of maltotriose and maltotetraose, thereby reducing their reactivity. Cyclodextrin is a macrocyclic oligosaccharide composed of six, seven, or eight α-D-glucopyranosyl units linked by α-1,4-glycosidic bonds, which can be applied to the regulation of more complex proteins [24]. Additionally, stachyose is a member of the raffinose family of oligosaccharides. Specifically, the 6-position of glucose is linked to α-D-galactose and 1,4-α-D-galactose [25]. This position corresponds to where the hydrogen bond, visualized during docking, connects with the stachyose small molecule.

*Lactobacillus* can interact with its external environment, resist environmental stress, and communicate with the host and intestinal flora through direct contact between its surface proteins and the surrounding environment. In other words, the surface proteins not only provide structural support and stability to the cell wall and membrane but also function as molecular sieves, recognizing polysaccharides and small peptides, and even acting as drug target receptors for drug delivery [26]. Additionally, these surface proteins can envelop and interact with the cell throughout the growth stages of *Lactobacillus* [27]. Consequently, surface proteins can screen, identify, capture, and engage with external molecules, thereby influencing or participating in various functions of *Lactobacillus*, including growth activity, acid tolerance, bile salt tolerance, gastrointestinal fluid tolerance, adhesion, and immune anti-inflammatory responses. The incorporation of nutritional small molecules and surface LPxTG proteins positively influences the growth activity of *L. reuteri*, aligning with the characteristic of probiotics to absorb and utilize prebiotics, thereby enhancing their probiotic effects. Rodríguez-Daza et al. [28] noted that certain probiotic strains possess enzyme libraries, such as tannase, α-L-rhamnosidase, and phenolic acid reductase, which are involved in converting various polyphenols into bioactive phenolic metabolites. In vivo studies have demonstrated that these (poly)phenol-transforming bacteria thrive when provided with phenolic substrates. It is speculated that polyphenols generally do not exhibit antibacterial effects, enabling competitive bacteria to occupy ecological niches and thereby promoting the proliferation of beneficial intestinal bacteria. Interestingly, phenolic small molecules such as rutin and myricetin can enhance the growth and reproduction of *L. reuteri*. Stachyose, a small oligosaccharide molecule, participates in the sugar metabolism of the strain and provides a carbon source, making its growth-promoting effect particularly significant. One study confirmed that stachyose can hydrolyze, inducing lactic acid bacteria (LAB) to produce more α-galactosidase, which in turn increases glucose metabolism and cell activity, thereby significantly promoting LAB proliferation [29]. Additionally, the combination of B3XKV5 with small molecules further enhances the growth of the strain, confirming that the surface protein can capture extracellular nutrient molecules and facilitate the strain’s absorption and utilization of nutritional factors from the external environment.

Surface proteins not only serve as structural support for the cell membrane and wall but also act as molecular sieves that can recognize polysaccharides, small peptides, and even function as drug-targeting receptors [26]. In the harsh intestinal environment, *Lactobacillus* can withstand external stress through various mechanisms, including transport systems, surface proteins, extracellular polysaccharides, and fatty acid synthesis [30]. Studies have shown that *L. reuteri* can absorb and utilize prebiotics while simultaneously exerting probiotic effects. Regarding gastrointestinal tolerance, the individual and synergistic additions of small molecules, proteins, phenols, and carbohydrates significantly impact strain tolerance, which correlates with the docking affinity. As a transmembrane protein, the surface layer LPxTG protein B3XKV5 participates in the transport, delivery, and even capture of molecules. A strong interaction allows small nutrient molecules to stably bind to B3XKV5, which is embedded in the cell membrane. With changes in the environment and stimuli, the protein undergoes conformational changes, facilitating the transfer of small molecules into the cell, where they are subsequently absorbed and metabolized. The qPCR results indicated that the gene expression of B3XKV5 in the gastrointestinal fluid environment was significantly higher than that in a normal environment, suggesting that the surface LPxTG protein is assembled in the cell membrane to enhance the strain’s tolerance in the gastrointestinal tract (GIT). Furthermore, as shown in Table S1, the circular dichroism analysis of the secondary structure revealed that B3XKV5 contains 37.6% α-helix, 26.6% β-sheet, and 8.8% β-turn structures. The addition of four small molecules altered the content of both the α-helix and β-sheet structures in B3XKV5. This finding indicates that the small molecules influence the conformation of B3XKV5, thereby confirming the interaction between these four small molecules and the LMP B3XKV5. This result aligns with the observations from gastrointestinal fluid tolerance and RT-qPCR analyses.

**Figure 7:**
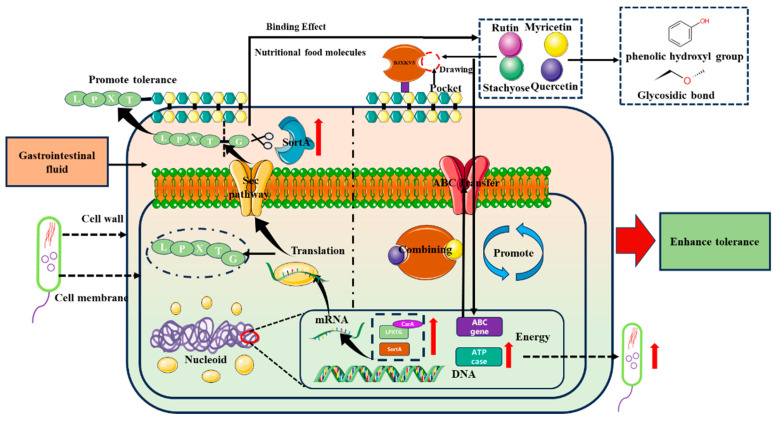
The mechanism of the interaction between small molecules and LMP B3XKV5 on the tolerance of *L. reuteri* in gastrointestinal fluid environment. The red arrow indicates the up-regulation of expression; black curved arrows indicate gene transcription and translation; black vertical up and down arrows indicate the delivery of matter.

Interestingly, the exogenous addition of B3XKV5 resulted in the inhibition of its own gene expression. Kurland [31] posits that the over-accumulation of bacterial proteins can trigger a hunger response. We infer that, while the exogenous addition of B3XKV5 may require increased secretion of the protein to combat external environmental stress under adverse conditions, sufficient levels of LMP led to the down-regulation of its gene expression. In a previous study, we analyzed the transcriptome of *L. reuteri* DSM 8533 under gastrointestinal fluid stress (Table S2). We observed that the genes involved in purine nucleotide metabolism were regulated in opposing directions, with those related to guanine and adenine biosynthesis (HN00_04545, purK, Lreu_0134, and purC) typically being up-regulated, potentially contributing to ATP accumulation [32]. The up-regulation of *carA*, which encodes an enzyme involved in aromatic amino acid biosynthesis, and *ilvB*, related to branched-chain amino acid biosynthesis, may provide protection against pH and bile invasion by generating hydrophobic regions [33,34]. Following culturing in gastrointestinal fluid, we noted an increase in the expression of the ABC transporter (Lreu23DRAFT_3567) and the ATP-binding component of the ABC transporter ATPase subunit (LBFF_1890), facilitating the exchange of intracellular and extracellular substances [35]. Based on the experimental results, we speculate that gastrointestinal fluid stress promotes the transcriptional up-regulation of related genes (Table S2), while the synergistic addition of B3XKV5 and small molecules significantly enhances B3XKV5 gene expression. The interaction between proteins and small molecules occurs either outside the cell or at the cell membrane, where small molecules are adsorbed and captured by the LPxTG motif proteins. The increase in ABC transporters and ATPase subunits enables the strain to utilize exogenously added proteins and small nutritional molecules to combat environmental stress (Figure 7). Furthermore, it has been confirmed that *Desulfovibrio vulgaris* can utilize nutrients to produce small molecules that influence the activity of AI-2 signaling molecules [36]. In this study, the addition of proteins and small nutritional molecules led to an increase in AI-2 signal intensity. This finding further confirms that bacterial surface proteins facilitate nutritional and informational exchanges between cells and their extracellular environment. Additionally, the results demonstrate that the interaction between small nutrient molecules and B3XKV5 enhances the biological utilization of these nutrients by the strain, resulting in the production of a greater quantity of signaling molecules.

## 5. Conclusions

In summary, this study revealed that the interaction between nutritional small molecules and bacteria surface protein B3XKV5 mediates the tolerance mechanism of *L. reuteri* to gastrointestinal fluid. As small molecules of intestinal nutrition, rutin, myricetin, quercetin, and stachyose contain special functional structures that can couple with the LMPs to synergistically promote the biological activity of *L. reuteri*, help it to colonize the intestine, and exert probiotic properties. In future studies, transcriptomics and metabolomics can be used to further explore the relationship between surface LPxTG protein–small molecule interaction and the tolerance mechanism of *L. reuteri*.

## Figures and Tables

**Table 1:** Modeling information of B3XKV5 protein in Swiss model.

Model	File	Built with	Oligo-State	Ligands	GMQE	QMEANDisCo Global
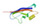	PDB	ProMod3 3.2.1	monomer	None	0.00	0.55 ± 0.08

**Table 2:** Modeling template information of B3XKV5 protein in Swiss model.

Template	Seq Identity	QSQE	Found by	Method	Resolution	Seq Similarity	Range	Coverage	Description
4ng0.1.A	38.30	0.00	HHblits	X-ray	1.50 Å	0.39	1772–1875	0.02	LAR_0958 cell surface adhension

**Table 3:** The docking affinity results and two-dimensional structures of phenolic nutrient small molecules.

Category	Name	Affinity (Kcal/mol)	Functional Group	2D Structure
phenols	Rutin	−6.5	Benzene ring, Phenolic hydroxyl	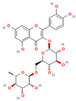
	Myricetin	−6.3	Benzene ring, Phenolic hydroxyl	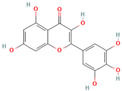
	Quercetin	−5.9	Benzene ring, Phenolic hydroxyl	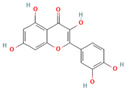
	Caffeic acid	−4.8	Phenolic hydroxyl, Carboxyl	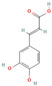
	Ferulic acid	−4.3	Phenolic hydroxyl, Carboxyl	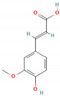

**Table 4:** The docking affinity results and two-dimensional structures of acid nutrient small molecules are represented.

Category	Name	Affinity (Kcal/mol)	Functional Group	2D Structure
acid	DL-3-Phenyllactic acid	−4.8	Benzene ring, Carboxyl	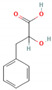
	Salicylic acid	−4.8	Benzene ring, Carboxyl	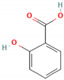
	Propanoic acid	−3.1	Carboxyl	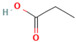
	Acetic acid	−2.7	Carboxyl	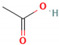

**Table 5:** Docking affinity results and two-dimensional structures of oligosaccharide nutritional small molecules.

Category	Name	Affinity (Kcal/mol)	Functional Group	2D Structure
Oligosacch-aride	Stachyose	−6.2	Glycosidic bond, Hydroxyl	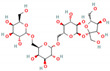
	Maltohexaose	−6.1	Glycosidic bond, Hydroxy	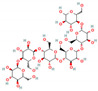
	Maltotetraose	−5.9	Glycosidic bond, Hydroxy	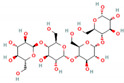
	Maltotriose	−5.9	Glycosidic bond, Hydroxy	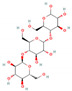
	Maltose	−5.0	Glycosidic bond, Hydroxy	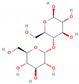
	Triose	−3.1	Hydroxy	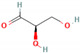

## Data Availability

The original contributions presented in the study are included in the article/Supplementary Materials, further inquiries can be directed to the corresponding author.

## Supplementary Materials

The following supporting information can be downloaded at https://www.mdpi.com/article/10.3390/foods13223685/s1, Table S1: Circular dichroism secondary structure analysis of protein B3XKV5 before and after binding to rutin, myricetin, quercetin, and stachyose; Table S2: Changes in related genes in *L. reuteri* under gastrointestinal fluid stress.
