# Supplementary material for: Coupled Effect of Nutritional Food Molecules and Lactobacillus reuteri Surface Protein Interaction on the Bacterial Gastrointestinal Tolerance
Source: Foods. 2024 Nov 19;13(22):3685. doi: 10.3390/foods13223685 (PMC11593405; doi:10.3390/foods13223685)
Supplement: Supplementary file 1 [file foods-13-03685-s001.zip › foods-3299793-supplementary.pdf]

Table S1. Circular dichroism secondary structure analysis of protein B3XKV5 before and after binding to rutin, myricetin, quercetin and stachyose

Name	α-helix (%)	β-sheet (%)	β-turn (%)	random coil (%)	RMS value
B3XKV5	37.6	26.6	8.8	27.0	5.248
Rutin-B3XKV5	41.9	20.9	11.3	25.9	8.770
Myricetin- B3XKV5	31.8	33.3	7.6	27.3	8.063
Quercetin- B3XKV5	40.6	27.7	5.2	26.6	5.491
Stachyose- B3XKV5	32.1	31.1	9.3	27.5	7.774

Table S2. Changes of related genes in *L. reuteri* under gastrointestinal fluid stress

Accession	Gene ID	Function	Log_2Fold change
tr|B3XKV5|	Lreu23DRAFT_4529	LPxTG-motif cell wall anchor domain protein	2.35
tr|R4RKB8|	LBFF_1890	ABC transporter ATP-binding component	1.58
tr|B3XRA5|	Lreu23DRAFT_3567	ABC transporter	3.19
tr|A0A073JMU6|	LR3_01370	Phosphate ABC transporter permease	3.18
tr|F8DNS1|	*pstB*	Phosphate import ATP-binding protein PstB	2.46
tr|A0A0G9GF74|	*carA*	Carbamoyl-phosphate synthase small chain	1.91
tr|A0A0N6WH84|	*ilvB*	Acetolactate synthase	1.47
tr|A0A098QUR7|	HN00_04545	Phage infection protein（Fragment）	5.79
tr|B3XNN2|	*purK*	N5-carboxyaminoimidazole ribonucleotide synthase	2.11
tr|A5VHT2|	Lreu_0134	5-(Carboxyamino）imidazole ribonucleotide mutase	2.12
tr|B3XNN4|	*purC*	Phosphoribosylaminoimidazole-succinocarboxamide synthase	4.27
